# A robust ensemble feature selection approach to prioritize genes associated with survival outcome in high-dimensional gene expression data

**DOI:** 10.3389/fsysb.2024.1355595

**Published:** 2024-03-20

**Authors:** Phi Le, Xingyue Gong, Leah Ung, Hai Yang, Bridget P. Keenan, Li Zhang, Tao He

**Affiliations:** 1 Division of Hematology/Oncology, Department of Medicine, University of California, San Francisco, San Francisco, CA, United States; 2 Department of Physiological Nursing, School of Nursing, University of California, San Francisco, San Francisco, CA, United States; 3 Helen Diller Family Comprehensive Cancer Center, University of California, San Francisco, San Francisco, CA, United States; 4 Department of Epidemiology and Biostatistics, University of California, San Francisco, San Francisco, CA, United States; 5 Department of Mathematics, San Francisco State University, San Francisco, CA, United States

**Keywords:** colorectal cancer, ensemble feature selection, high-dimensional data, time-to-event outcome, pseudo variables, group lasso

## Abstract

Exploring features associated with the clinical outcome of interest is a rapidly advancing area of research. However, with contemporary sequencing technologies capable of identifying over thousands of genes per sample, there is a challenge in constructing efficient prediction models that balance accuracy and resource utilization. To address this challenge, researchers have developed feature selection methods to enhance performance, reduce overfitting, and ensure resource efficiency. However, applying feature selection models to survival analysis, particularly in clinical datasets characterized by substantial censoring and limited sample sizes, introduces unique challenges. We propose a robust ensemble feature selection approach integrated with group Lasso to identify compelling features and evaluate its performance in predicting survival outcomes. Our approach consistently outperforms established models across various criteria through extensive simulations, demonstrating low false discovery rates, high sensitivity, and high stability. Furthermore, we applied the approach to a colorectal cancer dataset from The Cancer Genome Atlas, showcasing its effectiveness by generating a composite score based on the selected genes to correctly distinguish different subtypes of the patients. In summary, our proposed approach excels in selecting impactful features from high-dimensional data, yielding better outcomes compared to contemporary state-of-the-art models.

## Introduction

Next-generation sequencing (NGS) techniques (Hu et al., 2021) can provide us with information on the expression of more than 30,000 genes, which helps researchers understand gene regulations and interactions to find treatments for diseases. However, the number of genes associated with a particular disease is small (Yang et al., 2005). Therefore, we need to develop powerful tools to select genes that work as a group and are associated with clinical outcomes. Feature selection approaches were developed to choose the most relevant and informative features for research questions from the original raw set of features; therefore, they can help avoid overfitting, reduce training time, handle the challenge of dimensionality, and simplify data representations.

Survival analysis (Klein et al., 1992) is a statistical model studying time-to-event data in which the event may not be observed (censored) during the study because of loss to follow-up or early end of the study. Due to the presence of censoring, which is a unique characteristic in survival analysis, there is a need to develop novel techniques to work with feature selections for survival data, especially for high-throughput gene expression data in which most of the potential predictors are unimportant, with nearly no effect on the outcome (Friedman et al., 2010). The Cox proportional hazards model is the most commonly used technique for analyzing survival data. However, it was not designed for high-dimensional datasets with a large number of predictors. Lasso (Least Absolute Shrinkage and Selection Operator) introduces a penalty term to the Cox model’s likelihood function, which penalizes the absolute values of the regression coefficients. By forcing some coefficients to be exactly zero, Lasso effectively performing variable selection. In addition, there are models tailored to effectively handle situations where the number of features outweighs the number of observations (Li et al., 2018). Machine learning techniques that inherently handle high-dimensional data have been adapted to handle censored data, offering more flexible alternatives for analyzing high-dimensional, right-censored, heterogeneous data. However, unlike statistical models based on a mathematical framework, machine learning approaches do not impose a specified relationship on the predictors and outcomes and rely mainly on–data-driven algorithms, which makes it hard to interpret results. Furthermore, a lot of feature selection methods for survival analysis use a scoring model (Neums et al., 2019) to measure variations of features to select important features. Since the scoring algorithm was developed specifically to take care of the data censors and tie events of survival data, the results are biased (Munson et al., 2009) which may lead to selecting nonimportant features and provide a less accurate prediction.

We introduce a robust and effective “Pseudo-variables Assisted Group Lasso” method built on the ensemble idea, i.e., “more heads are better than one”, where features obtained from different selectors are aggregated to enhance the final selection. Moreover, we incorporated pseudo-variables which we know are irrelevant to the outcome and the permutation technique to assist the selection. The ensemble and pseudo-variables are nicely embedded into the Group Lasso model to yield the final output. Among aggregated features, only the features that consistently show stronger signals than the pseudo-variables (known noises) across permutations will be selected. We used colorectal cancer data from The Cancer Genome Atlas (TCGA) for illustration of our proposed approach. In addition, we performed simulation studies based on two different settings, where the first one mimicked the colorectal cancer data, and the second considered more complicated situations under various scenarios. For each simulation, we first simulated gene expression data for hundreds of genes and then generated survival outcomes based on some causal genes. The proposed feature selection ensemble method was applied to “uncover” the causal genes and compared to the existing methods.

## Materials and methods

### Colorectal cancer data set from TCGA database

Raw gene expression counts were downloaded from colon cancer (The Cancer Genome Atlas Network, 2012) datasets using The Cancer Genomics Cloud (Lau et al., 2017); additional clinical metadata was downloaded from cBioportal (Cerami et al., 2012). The mRNA-Seq data from TCGA was produced using the Illumina HiSeq 2000 platform and processed by the RNAseqV2 pipeline, which used MapSplice for alignment and RSEM for quantification.

### A robust feature selection ensemble

**FIGURE 1:**
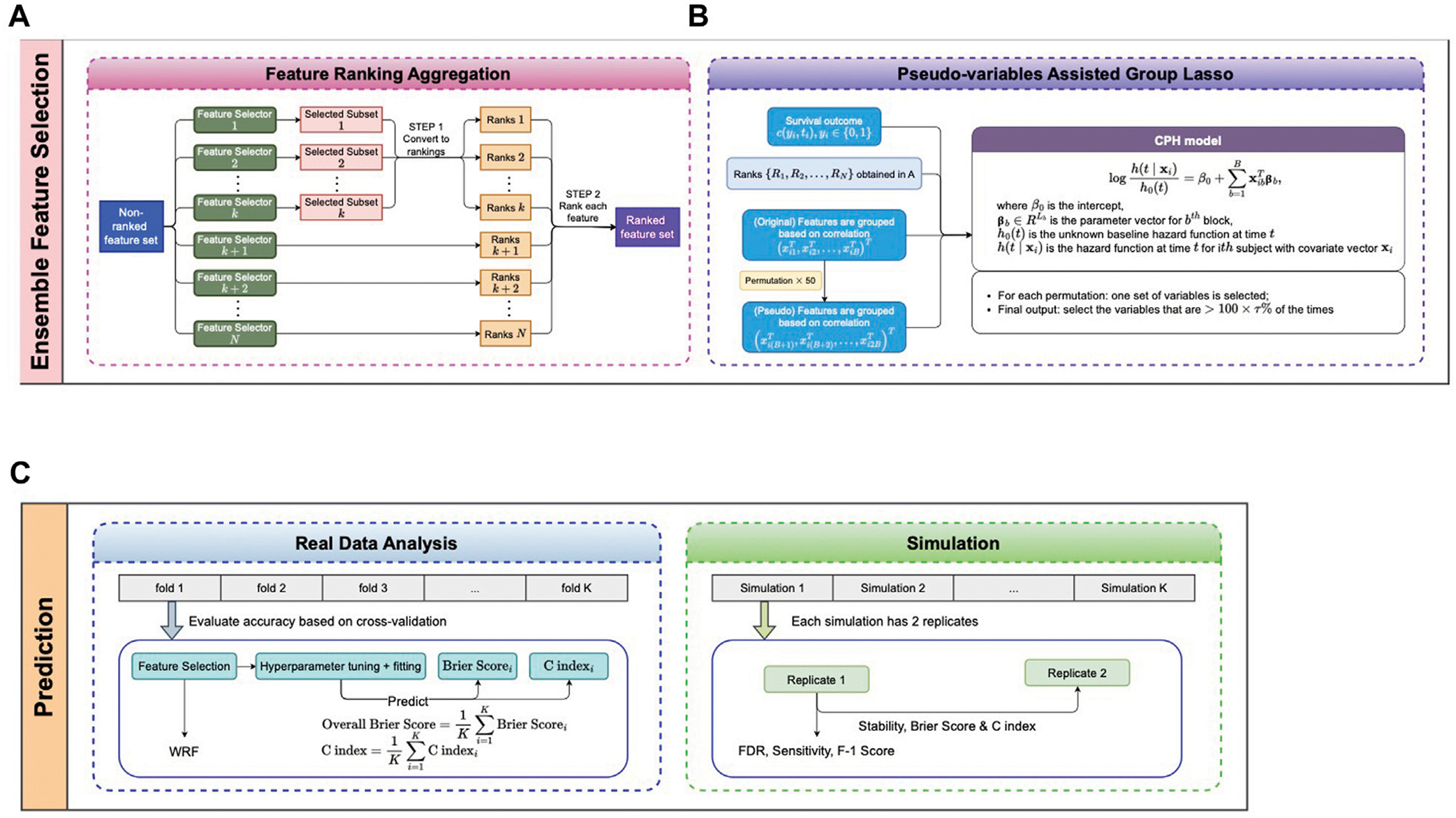
Proposed pipeline. **(A, B)** Ensemble feature selection. **(A)** Feature selection based on different methods and aggregation of selected features. **(B)** Pseudo-variables assisted group lasso. **(C)** Prediction for real data and simulation datasets.

**FIGURE 2:**
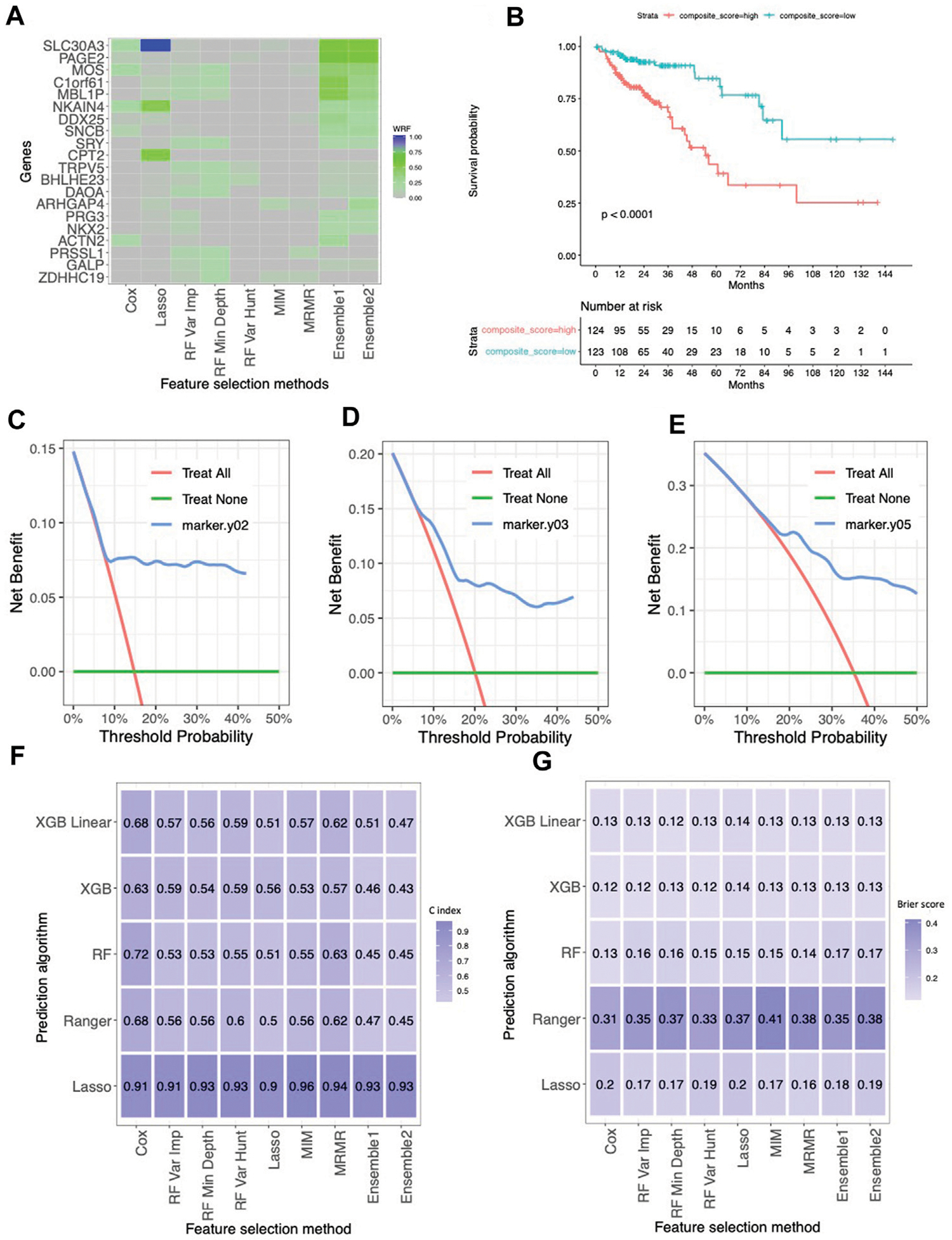
The results for the TCGA colorectal cancer dataset. **(A)** Normalized selection frequency of the top 20 selected genes by each feature selection approach. Each row represents an individual single gene, and each column represents the feature selection approaches. **(B)** Kaplan-Meier survival curves. The low-risk group and high-risk group were defined by median of the composite score. The composite score was calculated as the linear combination of those genes selected by ensemble approach and their coefficients in the cox proportional hazard model. **(C–E)** DCA of 2 year, 3 year and 5 year. **(F)** Heatmap of concordance index (C-index). The heatmap shows the mean value of the C-Index across 5 repeats of 5-fold cross-validation for each combination of machine learning algorithms (rows) and feature selection methods (columns). **(G)** Heatmap of Brier Score. The heatmap shows the mean value of the Brier Score across 5 repeats of 5-fold cross-validation for each combination of machine learning algorithms (rows) and feature selection methods (columns).

The proposed pseudo-variable-assisted feature ensemble procedure has two major steps: 1) aggregating the feature selection results from multiple feature selectors (Figure 1A) and 2) fitting a group Lasso model on the identified feature set with a new permutation-assisted tuning strategy (Figure 1B). In the second step, the group is defined based on the correlation structure, ensuring that features are highly correlated within each group.

Aggregating the results from different feature selection approaches is a critical step in ensemble learning. The outputs of the different approaches can be various, either the subsets of selected features, the rankings of all features, or both. We applied the same scheme as in (He et al., 2022) to obtain the ranked feature set depending on the types of outputs (Figure 1A), where the final rank is an aggregation from each ranking. We assume that the observations are $\left(x_{i},y_{i}\right),i=1,\ldots ,n$, where $x_{i}$ is a G-dimensional vector in which each feature has its aggregated rank, and $y_{i}$ is a survival outcome. Without loss of generality, we assume the G features are quantitative variables (e.g., gene expressions). However, the proposed method can be applied to categorical or mixed-type variables. Similar to (He et al., 2022), we can rewrite the G-dimensional vector $x_{i}$ as $x_{i}={\left(x_{i1}^{T},x_{i2}^{T},\ldots ,x_{iB}^{T}\right)}^{T}$ with $x_{ib}$ of dimension $L_{b},b=1,\ldots ,B,\;\sum_{b=1}^{B}L_{b}=G,$ based on their correlation structure such that within each block, the absolute value of pairwise correlation is all greater than a correlation threshold $\rho_{T}$.

Next we consider a Group Lasso model (Utazirubanda et al., 2021) on the ranked feature set (Figure 1B) for survival outcomes. For commonly seen right censored survival data, $y_{i}=\left({\text{T}}_{\text{i}},\Delta_{i}\right)$ is a survival outcome, where $T_{i}=\text{min}\left({\text{U}}_{\text{i}},V_{i}\right),\Delta_{i}=I\left(U_{i}\leq V_{i}\right)\in \{0,1\}$, with $U_{i}$ and $V_{i}$ denote the event time of interest and the censoring time for the i th subject, respectively. We model the relationship between the survival outcomes $y_{i}$ and features $x_{i}$ using the Cox proportional hazards model (Deo et al., 2021)

$$log\frac{h\left(t|x_{i}\right)}{h_{o}(t)}=\beta_{0}+\sum_{b=1}^{B}x_{ib}^{T}\beta_{b}≜\gamma_{\beta }\left(x_{i}\right),$$

where $\beta_{0}$ is the intercept, and $\beta_{b}\in R^{L_{b}}$ is the parameter vector for the bth block, $h_{o}(t)$ is the (unknown) baseline hazard function at time t, and $h\left(t|x_{i}\right)$ is the hazard function at time t for the ith subject with covariate vector $x_{i}$. We aim to identify which gene groups amongst the B groups associated with the survival outcomes.

Based on the partial likelihood function,

$$L(\beta )=\prod_{i=1}^{n}{\left\{\frac{\text{exp}\left[\gamma_{\beta }\left(x_{i}\right)\right]}{\sum_{k\in Q_{j}}\text{exp}\left[\gamma_{\beta }\left(x_{k}\right)\right]}\right\}}^{\Delta_{i}},$$

$Q_{j}=\left\{k:T_{k}\geq T_{j}\right\}$, we can obtain the estimation of the complete parameter vector β by minimizing the following objective function.


$$Q_{\lambda }\left(\beta \right)=-L\left(\beta \right)+\lambda \sum_{b=1}^{B}s_{b}{\left\|\beta_{b}\right\|}_{2}.$$

Recall that λ is the tuning parameter that controls the amount of shrinkage (larger λ shrinks more coefficients to zero), and $s_{b}$ is used to rescale the penalty to each group. To ensure the top-ranked features are more likely to be selected, we put a small penalty on top-ranked feature sets by proposing using the product of the minimum rank among each feature set and $\sqrt{L_{b}}$.

The objective of this study is more about selecting the important features than improving the prediction accuracy. Therefore, we propose to use the pseudo-variables assisted tuning strategy (He et al., 2022) to facilitate the group-lasso tuning parameter selection. This strategy is built on the idea of combining the original and permutated input features (e.g., expressed genes), where the permutations work as a control to determine the significance of each group. Hence, we can select significantly important genes (not by chance).

It is known that the λ in group-lasso-type regularization controls the amount of shrinkage. As λ increases, fewer groups are selected. A group can be considered more important one if it is selected when λ is large. Based on these observations, we can define an importance measure $V_{b}=\text{sup}\{\lambda$: the coefficient for bth group is nonzero}, for each of the 2B groups, including the B groups from original input features (b=1,…,B) and their B groups of permutated copies (b=B+1,…,2B). For each permutation, groups from original input features are selected if their $V_{b}$ is larger than $\underset{B+1\leq b\leq 2B}{\text{max}}V_{b}$, i.e., the strongest signal among permutated groups which we have known are irrelevant groups. After running K (e.g., K = 50) times of permutations, we selected the groups of features that have been selected more than a certain number of percentages τ (i.e., τ=0.5) among K permutations.

### Feature selection and machine learning algorithms

In our study, we evaluated nine different feature selection methods, including seven existing feature selection methods and two robust ensemble feature selectors we constructed. The nine selectors can be divided into four major groups: (I) feature selection algorithms based on mutual information optimization: mutual information maximization (MIM) (Torkkola, 2003), minimum redundancy maximum relevance (MRMR) (Radovic et al., 2017); and (II) random forest-based approaches: a random forest minimal depth (RF Min Depth) (Ishwaran et al., 2008; Ishwaran et al., 2011), a random forest variable importance (RF Var Imp) (Archer and Kimes, 2008), a random forest variable hunting (RF Var Hunt) (Chen and Ishwaran, 2013); and (III) Cox-based approaches: Cox hazard proportional (Cox) (Deo et al., 2021) and $\ell_{1}$ penalized Cox (Lasso) (Goeman, 2010); (IV) ensemble learners (Zhou, 2021). We created two feature ensembles, Ensemble 1 and Ensemble 2, where the first one is the ensemble of Lasso, Cox, and MIM, and the second is the ensemble of Lasso, Cox, MIM, and MRMR. Parameters used in the paper were included in Table 1.

To compare the results of our feature selection ensemble method with others, we tested the selected features on five well-known prediction models using machine learning and non-parametric techniques: (I) the Cox model with $\ell_{1}$ regularization (Lasso) (Binder, 2015); (II) models based on boosted trees: xgboost (XGB) (Chen and Guestrin, 2016) (III) boosted gradient linear models: xgboost based on linear learner (XGB linear) (Chen and Guestrin, 2016) and (IV) random forest-based methods: random survival forest (RF) (Segal, 2004) and ranger (Wright and ranger, 2017). All feature selection methods and machine learning algorithms assessed here can handle the time-to-event outcome.

### Simulation

To mimic the correlation structure in real data, we conducted a simulation based on the colorectal cancer data. Considering in the real world, we usually do not often observe the causal variables directly, but rather the variables that are highly correlated with the causal variables, if any. Here we use a modified version of the simulation strategy as in (Degenhardt et al., 2019; He et al., 2022) to mimic this real-world situation. We first picked six correlated gene expression blocks from the colorectal cancer data, where each block included 6,7,8,7,8 and 9 highly correlated genes (correlation coefficient greater than 0.5) respectively (Supplementary Table S1). For each of the first three blocks, we randomly selected one of the genes as the unobserved causal variables ($z_{1},{\text{z}}_{2}$, and $z_{3}$) which are in the boldface in Supplementary Table S1 and the rest of the genes in the first three blocks as observed causal variables $\left\{v_{i}^{\left(j\right)},i=1,2,3;\text{j}=1,\ldots ,{\text{J}}_{i}-1,J_{1}=6,J_{2}=7,J_{3}=8\right\},$ while considering the genes from the last three blocks $\left\{v_{i}^{\left(j\right)},i=4,5,6;\text{j}=1,\ldots ,{\text{J}}_{i},J_{4}=7,J_{5}=8,J_{6}=9\right\}$ as observed noncausal variables. For i th block, the variables $\left\{v_{i}^{\left(j\right)},J=1,\ldots ,J_{i}\right\}$ were generated using multivariate normal distribution with mean zero and the correlation matrix computed based on the real data. Then we generated survival outcomes using the three unobserved causal variables based on a Cox proportional hazards model using the *reda* R package (Fu et al., 2022) (*simEvent* function), with $h_{o}(t)$ set as 1,

$$log\frac{h(t|z)}{h_{o}(t)}=\beta_{0}+\beta_{1}z_{1}+\beta_{2}z_{2}+\beta_{3}z_{3}$$

In addition, we generated G-42 independent predictor variables $w_{k},k=1,\ldots ,G-42$, which are uncorrelated with the base variables $\left\{v_{i}^{\left(j\right)}\right\}$, are simulated based on a uniform distribution of (0,1). The input G=1000 features consisted of $\left\{v_{i}^{\left(j\right)},i=1,2,3;\text{j}=1,\ldots ,{\text{J}}_{i}-1,J_{1}=6,J_{2}=7,J_{3}=8\right\},\left\{v_{i}^{\left(j\right)},i=4,5,6;\text{j}=1,\ldots ,{\text{J}}_{i},J_{4}=7,J_{5}=8,J_{6}=9\right\}$ and $\left\{w_{k},k=1,\ldots ,G-42\}\right.$. We generated paired replicates (two n×G matrixes) with the first used for feature selection evaluation and the prediction models training, and the second used for assessing stability of feature selection and evaluating the prediction performance, and we repeated the processes for 100 times. The details of this real-data-based simulation, including the coefficients, full list of the gene blocks, and names of the unobserved causal genes, are provided in Supplementary Table S1. For ease of presentation, we will refer this real-data-based simulation as Simulation A below.

To further evaluate the performance of the proposed method under more diverse scenarios, we performed additional simulations (referred as Simulation B below). Similar to Simulation A, we first generate unobserved causal variables ($z_{1},{\text{z}}_{2}$, and $z_{3}$) and then the observed variables, where some are highly corrected with the causal variables (i.e., observed causal variables), and the rest are irrelevant (i.e., noise variables). The survival outcome is also simulated based on a Cox proportional hazards model using the *reda* R package (Fu et al., 2022) (*simEvent* function)

$$log\frac{h(t|z)}{h_{o}(t)}=\beta_{1}z_{1}+\beta_{2}z_{2}+\beta_{3}z_{3}$$

where $h_{o}(t)$ is set as 1. The three base variables ($z_{1},{\text{z}}_{2}$, and $z_{3}$, the unobserved causal variables) and three additional independent base variables ($z_{4},z_{5}$ and $z_{6}$, the unobserved non-causal variables) are independently sampled from a uniform distribution of (0,1). For each of the base variables $z_{i}$, we generate a set of 10 correlated predictor variables $v_{i}^{\left(j\right)}$, denoting the j th variable in group i, for j=1,…,10 and i=1,…,6, using the following formula:

$$v_{i}^{\left(j\right)}=z_{i}+\left(0.01+\frac{0.5(j-1)}{9}\right)\times N\left(0,0.3\right),$$

The correlation between the base variable $z_{i}$ and $v_{i}^{\left(j\right)}$ decreased as j increased. Please note that $z_{i},i=1,\ldots ,6$, are only used to simulate correlated variables $v_{i}^{\left(j\right)}$, and are not included for feature selection and classification. G-60 independent predictor variables $w_{k},k=1,\ldots ,G-60$, which are uncorrelated with the base variables $\left\{v_{i}^{\left(j\right)}\right\}$, are also simulated based on a uniform distribution of (0,1). Here we assume that the base variables are not observed. Hence, the input features consist of 30 observed causal variables $\left\{v_{i}^{\left(j\right)},i=1,2,3;\text{j}=1,\ldots ,10\right\}$ and 30 correlated, non-causal variables $\left\{v_{i}^{\left(j\right)},i=4,5,6;\text{j}=1,\ldots ,10\right\}$ and G-60 uncorrelated, non-causal variables $\left\{w_{k},k=1,\ldots ,G-60\right\}$, a total of G variables.

We consider twelve different simulation scenarios (Table 2) including 1) different event rates (η=0.3,0.5,0.7) which are mainly determined by the coefficients in the Cox proportional hazards model; 2) sparsity of causal genes (2.5%, 5%) with a different number of genes (G = 600 and 1,200); and 3) different sample sizes (n = 100 and 200). Similar as in Simulation A, for each of the scenarios, we generated 100 paired replicates, where each pair is consisted of two n×G matrixes.

### Model evaluation

In the real data studies, the causal variables are unknown. Moreover, due to different algorithm, we may have different lists of selected features across all methods. Therefore, to determine the important rank of features, we proposed using a weighted relative frequency (WRF) to measure the relative frequency that a feature is selected across five different folds as in (He et al., 2022). The weight of each selection is reciprocal to the number of features selected, i.e., larger set of selection adds less weight to each selected feature. A higher WRF indicates this feature is more consistently and sparsely selected across different folds.

Since the causal variables are known in simulation studies, we can evaluate the feature selection performance by comparing the selection to the truth (the known causal variables). Specifically, we used the following four commonly used metrics: false discovery rate (FDR), sensitivity, stability, F-1 score and empirical powers. FDR is the proportion of false-positive features in the selected feature set. Sensitivity is calculated as the proportion of selected causal variables among all the causal variables. Stability is calculated using Jaccard’s index: the ratio of the length of the intersection and the length of the union of two sets, where the two sets are the selections from the paired replicates. F-1 score is calculated as $2\frac{\text{precision}*\text{sensitivity}}{\text{precion}+\text{sensitivity}}$, serving as a balanced metric (harmonic mean) between sensitivity and precision (1-FDR). Empirical power could be calculated for each of the causal variables. It is the ratio that this particular causal variable is selected among the simulation replicates. A power of 1 indicates this casual variable was identified in each replicate, and a power of 0 means it was never selected across replicates. For each feature selection method and each of the twelve scenarios, we reported the average FDR, sensitivity, stability, F-1 score, and empirical powers across the first replicate of each of the 100 simulations.

Furthermore, to check the effectiveness on the predictions of our selected features compared to other well-known models, we used the Integrated Brier score (Ishwaran et al., 2008; Moradian et al., 2017) to assess the accuracy of predicted survival probabilities over a specified time period of events. Lower values of the Integrated Brier Score indicate better predictive accuracy, with 0 being the optimal score (perfect prediction) and 1 representing a model with no predictive ability. Harrell’s C-statistic, also known as the concordance index (C-index), was used to evaluate discrimination with a higher value indicating better discrimination, meaning the model is better at distinguishing between different outcomes. A C-index of 0.5 suggests that the model’s predictions are no better than random chance, while a C-index of 1.0 indicates perfect discrimination.

## Results

### Key features selected by the ensemble feature selection approach on a colorectal cancer (CRC) dataset

In the cohort of n=253 colorectal cancer subjects, encompassing 19,947 genes, the median overall survival (OS) was 83.2 months, with a median follow-up time of 22.5 months. We identified G=2,303 genes with p-value less than 0.05 based on the univariable Cox proportional hazard model to further evaluate different feature selection and prediction approaches. We then applied our proposed ensemble approach, where the groups were defined based on the correlation structure of the G=2,303 genes, such that within each block, the absolute value of pairwise correlation is all greater than 0.75. The proposed ensemble approaches (Ensemble 1 and Ensemble 2) show the consistency of selected genes and their important rankings compared to all genes, while other methods can only recognize some of them based on WRF (Figure 2A). Notably, the gene *SLC30A3*, although selected by Lasso with the highest WRF, was not identified by other methods. However, it attained the top rank in our proposed ensemble approach, showing the strength of the ensemble approach. Conversely, several genes (*MOS, C1ORF61*, and *MBL1P*) that did not rank highest in Lasso achieved top positions in random forest approaches, contributing to higher WRF in the ensemble approaches. Within the top five genes based on WRF (Ensemble 1 and Ensemble 2), *SLC30A3, MOS, C1ORF61*, and *MBL1P* genes were found to have an association with CRC (Lin et al., 2007; Zheng et al., 2015; Yin et al., 2020; Peng et al., 2021; Cui et al., 2022), gene *PAGE2*, a gene from cancer-germline genes, was found to be upregulated in Caco-2 colorectal cancer cell line (Yilmaz-Ozcan et al., 2014). On the other hand, other methods identified some of the above genes that has connections with colorectal cancers. Using these five genes, we created a composite score by calculating a linear combination of the gene expressions multiplied by their respective coefficients in a multivariable Cox proportional hazards model. Figure 2B presents the Kaplan Meier curves for the subjects with a composite score above and below the median composite score (which is −0.40), with a median OS of 54.6 months and not reachable (log-rank test p-value <0.001), respectively. The DCA (Decision Curve Analysis) curves based on 2 years, 3 years and 5 years (Figures 2C–E) consistently show that the net benefit curve outperforms reference lines across various threshold probabilities, indicating clinical utility. As shown in C-index (Figure 2F) and Brier scores (Figure 2G), in general, the prediction approaches have the most impact on the prediction performance rather than the feature selectors. Lasso has a higher C index, and random forest, XGB, and XBG linear yield the lowest Brier scores, while Ranger demonstrates relatively poorer performance.

### Improved performance by the ensemble feature selection approach based on simulation studies

**FIGURE 3:**
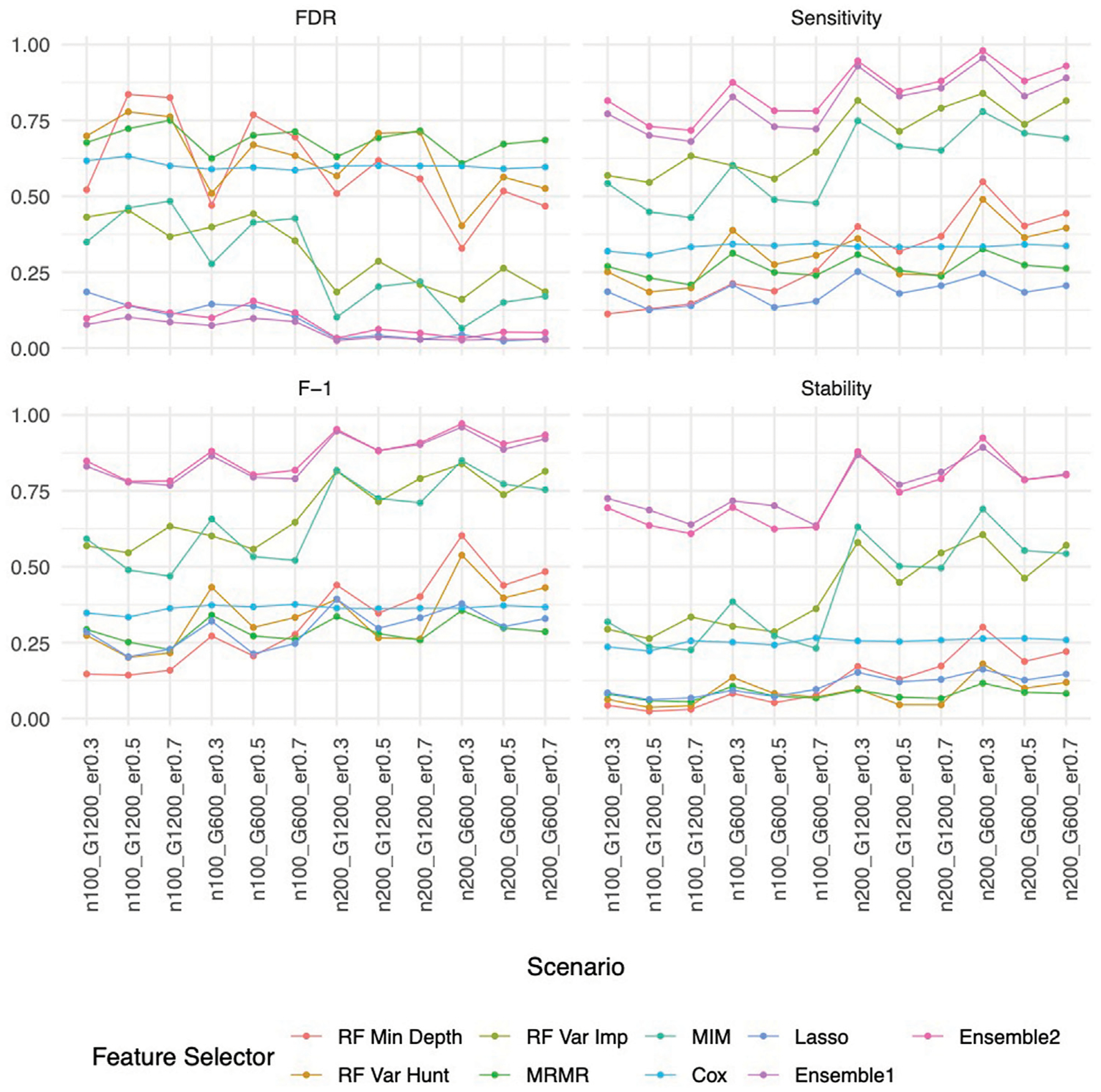
Feature selection performance based on Simulation B. In each panel, x-axis stands for different simulation listed in Table 1, y-axis stands for different evaluation metrics including FDR, Sensitivity, F-1 and Stability. For example, n100_G1200_eta0.3 stands for sample size is 100 with 1,200 candidate genes and the event rate is 0.5. Each colored curve stands for different feature selection approaches.

**FIGURE 4:**
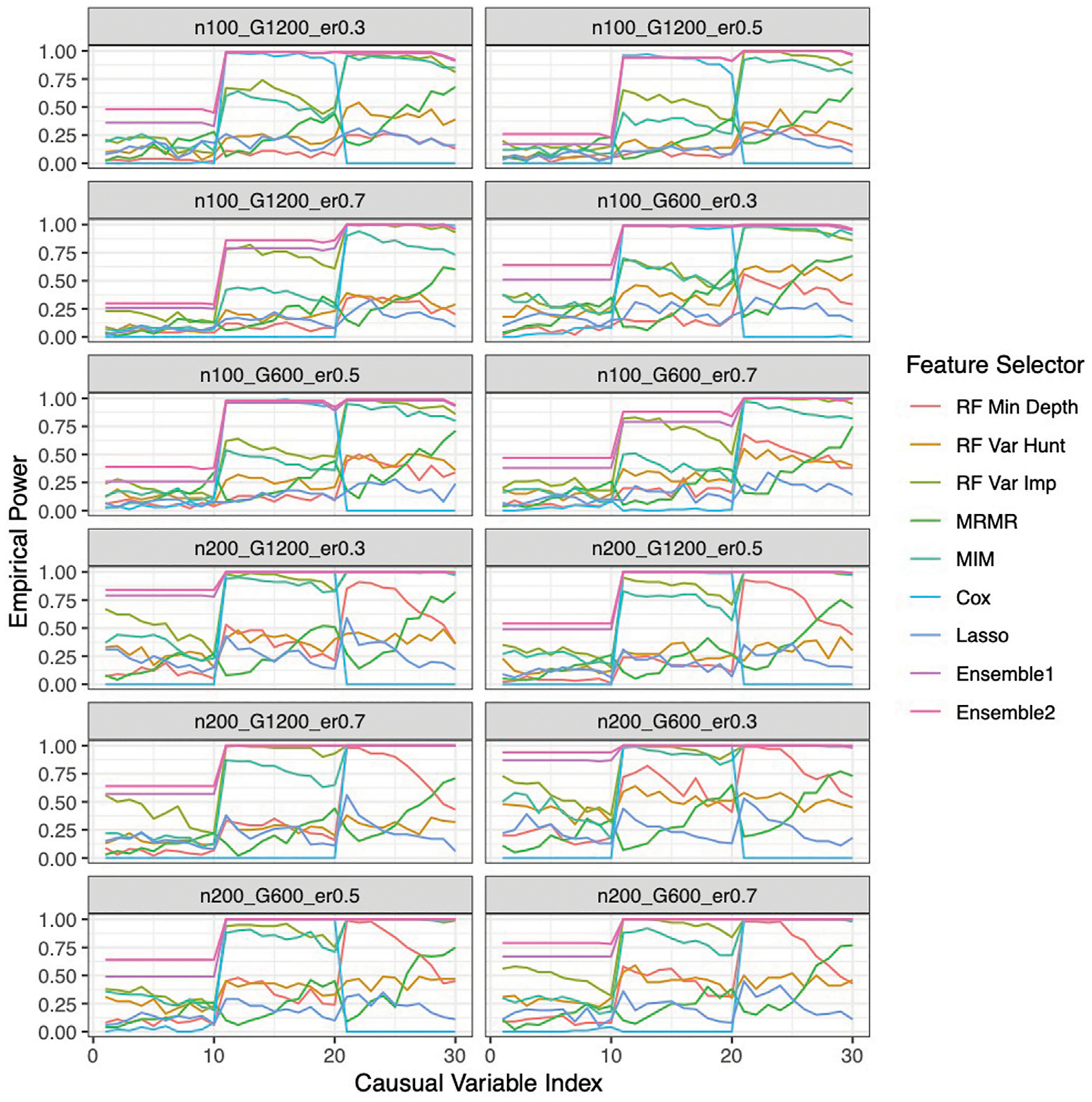
Empirical power of the feature selection approaches based on Simulation B. Each panel represents different simulation scenario listed in Table 1. For example, n100_G1200_eta0.3 stands for sample size is 100 with 1,200 candidate genes and the event rate is 0.5. In each panel, x-axis stands for the causal variable index.

Our ensemble approaches consistently demonstrated superior feature selection performance compared to other methods (Supplementary Figure S1A; Figure 3) with Ensemble 1 and Ensemble 2 exhibiting similar performance based on both Simulation A and Simulation B. Although the Lasso method also had low FDRs, it had the lowest sensitivity, reduced F-1 and lower stability. The random forest approaches overall showed poor performance. As expected, in general, a larger sample size (200 vs. 100) resulted in improved performance for all feature selection approaches. However, the impact of the gene sparsity of (2.5% vs. 5%) and event rates (0.3, 0.5, 0.7) on prediction performance was minimal, with slightly better performance observed at lower sparsity. In Supplementary Figure S1B; Figure 4, the empirical power of our ensemble approaches is consistently higher than or at least equivalent to that of other feature selectors across all thirteen scenarios (1 scenario for simulation A, and 12 for simulation B) for all 30 causal variables.

**FIGURE 5:**
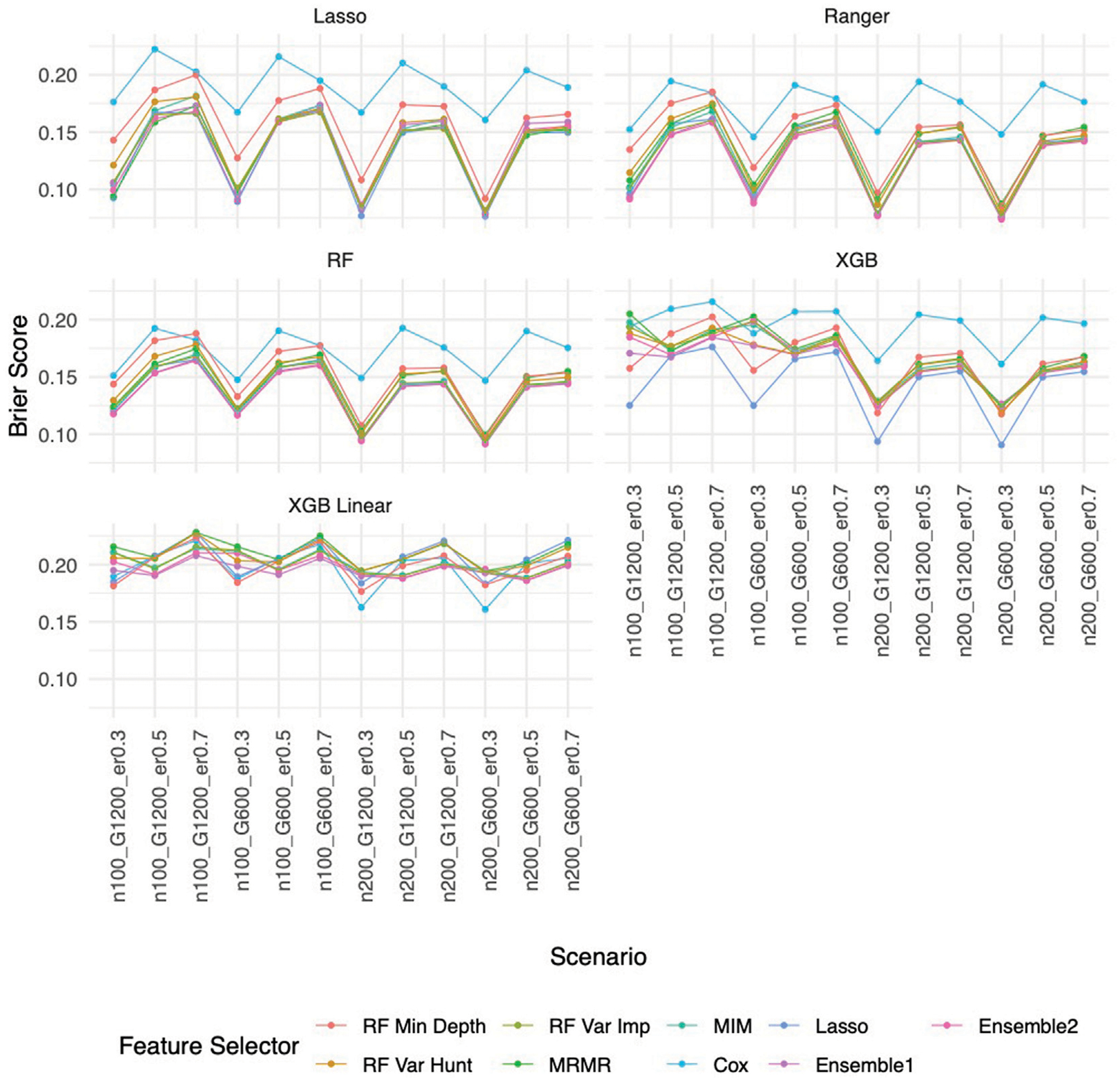
Brier score based on Simulation B. Panels present the Brier score for the corresponding prediction approach as indicated. In each panel, x-axis stands for different simulation scenario listed in Table 1. For example, n100_G1200_eta0.3 stands for sample size is 100 with 1,200 candidate genes and the event rate is 0.5. Each colored curve stands for different feature selection approaches.

**FIGURE 6:**
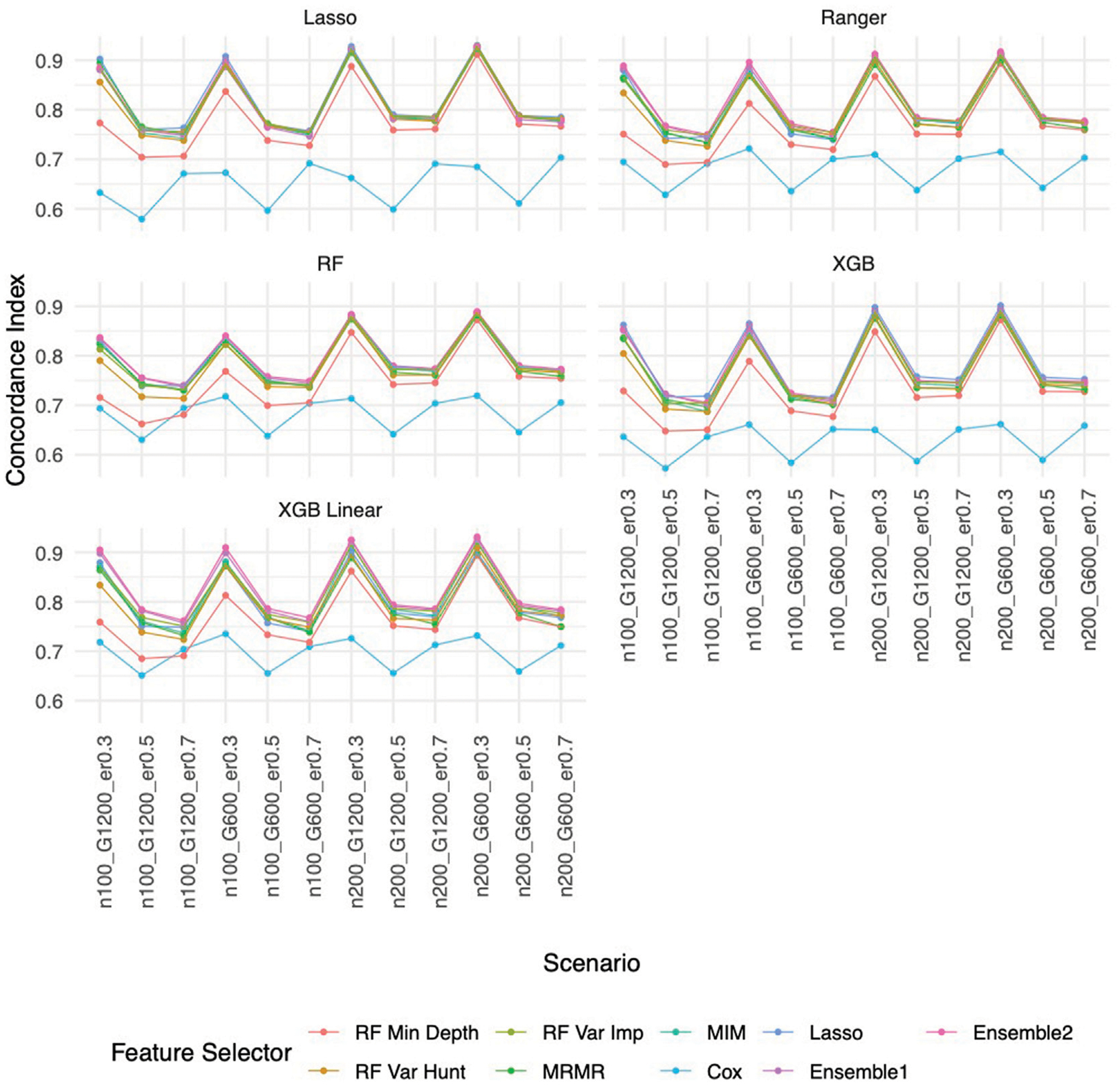
C-index based on Simulation B. Panels present the C-index for the corresponding prediction approach as indicated. In each panel, x-axis stands for different simulation scenario listed in Table 1. For example, n100_G1200_eta0.3 stands for sample size is 100 with 1,200 candidate genes and the event rate is 0.5. Each colored curve stands for different feature selection approaches.

Similar to the real data analysis, the overall impact on prediction performance is predominantly driven by the choice of prediction approaches rather than the feature selectors due to models’ bias. This observation is expected, as feature selection does not guarantee an improvement in prediction performance. Nevertheless, feature selection proves valuable by reducing the dimensionality and complexity of predictive models, leading to quicker model training times and improved convergence. Predictably, across all prediction approaches, feature selection based on the univariate Cox proportional hazards model consistently exhibited the least favorable performance, while the various selector approaches appeared quite similar. Notably, a higher event rate corresponded to larger Brier scores (Figure 5) and smaller C-index (Figure 6), indicating poorer prediction performance. A larger sample size contributed to slightly improved prediction performance in terms of Brier score and C-index. Interestingly, gene sparsity did not exert a notable impact on prediction performance. While our feature selection models may not have surpassed others in terms of accuracy measurements, we observed that they provided a stable and consistent accuracy across all measurements (as shown in Figure 5, 6; Supplementary Figures S1C, S1D). This suggests that the features we selected are significant and exhibit less bias, contributing to the reliability of our selected features. We also performed Simulation C with smaller effect sizes (Supplementary Table S2) with the same setting as Simulation B. The results (Supplementary Figures S2–S4) were consistent with all the observations mentioned above.

## Conclusion and discussion

This paper proposes a robust ensemble feature selection approach tailored explicitly for survival analysis. The ensemble feature selection approach is built on enhancing the feature selection process by combining different feature selection algorithms, ultimately improving the quality of feature selection and providing stabilized results. This is accomplished through a novel ranking algorithm integrated with a group lasso model, which is particularly advantageous when dealing with feature groups. Therefore, our proposed model is well-suited for applications in genetic data studies, where it is imperative to analyze genes as cohesive groups rather than individual entities. The proposed approach demonstrates a unique ability to select the most compelling features from top-tier models.

The key benefits of ensemble feature selections are 1) Robustness: by aggregating the results from diverse feature selection methods, the final ensemble is less likely to be influenced by the biases or limitations of a single feature selector; 2) Improved Generalization: the ensemble of multiple feature selectors, each built on a different algorithm, can lead to improved generalization and better performance on unseen data; 3) Model Agnosticism: feature selection ensembles are usually model-agnostic, meaning it is not tied to or dependent on a specific machine learning model. Instead, they can be applied across various feature selection models without favoring one over the other, making them widely applicable.

Though we only applied the proposed method to gene expression data, our method can be applied to a wide variety of data having very large number of features in genetics/genomics studies and medical research in general, such as genomic data, transcriptomic data, epigenomic data, proteomic data, clinical and phenotypical data and so on. Besides, the proposed method can smoothly take care of the correlated structure, and even utilize the natural set from certain biological knowledge such as pathway. Moreover, ensemble feature selection can be applied to different response variable, including quantitative, qualitative and time-to-event responses. Although the prediction gain is incremental, the benefits of feature selection are still significant. Firstly, it can enhance the interpretability particularly in the biomedical field and aims the discovery of meaning biological insights. Secondly, it can greatly improve the computational efficiency of downstream analysis, making it more feasible to handle large-scale data sets. Thirdly, it can help filter out irrelevant noise variable, avoid overfitting and enhance the reliability of the analyses.

## Supplementary Material

Table 1

Image 1

## Figures and Tables

**TABLE 1:** Parameters used for feature selection methods.

Approach	R package	Parameter	Description	Value
MIM (select top k)	praznik	k	Select top k features	25
MRMR (select top k)				
RF Min Depth (select top k)	randomForestSRC	ntree	Number of trees	1,000
RF Var Imp (select top k)		mtry	Number of variables to possibly split at each node	default
		nodesize	Minimum size of terminal node	15
RF Var Hunt (select top k)		k	Select top k feature	25
		nsplit	Number of random splits for splitting a variable	10
Cox (select up to top k which have p-value less than α)	survival	k	Select top k feature	25
		alpha	p-value threshold	0.05
LASSO	glmnet	lamba	Tuning parameter grid values	10^(−10,−9.9,…,0,…,9.9,10)^
Ensemble1		$\rho_{T}$	Minimum pairwise correlation within block	0.75
Ensemble2		K	Total number of permutations	50
		τ	Threshold of selection percentage	0.5

**TABLE 2:** Simulation scenario.

Scenarios	Label	Sample size	# Of genes	Event rate	Sparsity (# of causal genes/# of genes)	$\beta_{1}$	$\beta_{2}$	$\beta_{3}$
1	n100_G1200_er0.3	100	1,200	0.3	30/1,200	−6	8	−10
2	n100_G1200_er0.5	100	1,200	0.5	30/1,200	−2	3	−4
3	n100_G1200_er0.7	100	1,200	0.7	30/1,200	−2	−3	4
4	n100_G600_er0.3	100	600	0.3	30/600	−6	8	−10
5	n100_G600_er0.5	100	600	0.5	30/600	−2	3	−4
6	n100_G600_er0.7	100	600	0.7	30/600	−2	−3	4
7	n200_G1200_er0.3	200	1,200	0.3	30/1,200	−6	8	−10
8	n200_G1200_er0.5	200	1,200	0.5	30/1,200	−2	3	−4
9	n200_G1200_er0.7	200	1,200	0.7	30/1,200	−2	−3	4
10	n200_G600_er0.3	200	600	0.3	30/600	−6	8	−10
11	n200_G600_er0.5	200	600	0.5	30/600	−2	3	−4
12	n200_G600_er0.7	200	600	0.7	30/600	−2	−3	4

## Data Availability

Publicly available datasets were analyzed in this study. This data can be found here: https://portal.gdc.cancer.gov/projects/TCGA-COAD.
