# Supplementary material for: A robust ensemble feature selection approach to prioritize genes associated with survival outcome in high-dimensional gene expression data
Source: Front Syst Biol. 2024 Mar 21;4:1355595. doi: 10.3389/fsysb.2024.1355595 (PMC11786965; doi:10.3389/fsysb.2024.1355595)
Supplement: Supplementary file 1 [file Table1.DOCX]

**Table S1: Simulation Study A Setting**

| Block | Genes (unobserved causal genes is in bold, if any) | Coefficient of the causal gene |
| --- | --- | --- |
| 1 | NAT1.9, FDFT1.2222, GSR.2936, PPP2CB.5516, **KCTD9.54793**, MTUS1.57509 | $\beta_{1}= -1.5$ |
| 2 | **CLCN3.1182**, G3BP2.9908, ARFIP1.27236, GALNT7.51809, USP53.54532, USP38.84640, SH3D19.152503 | $\beta_{2}= -0.5$ |
| 3 | **BCAT1.586**, TNFAIP6.7130, TWIST1.7291, DPYSL4.10570, POSTN.10631, MAGED4B.81557, NKAIN4.128414, MAGED4.728239 | $\beta_{3}=1$ |
| 4 | BNC1.646, UCHL1.7345, SRPX.8406, LRRN2.10446, HCG4.54435, TCEAL5.340543 | NA |
| 5 | CPT2.1376, FUCA1.2517, HMGCL.3155, PAFAH2.5051, PXMP2.5827, NMNAT1.64802, DHDDS.79947 | NA |
| 6 | GRSF1.2926, HADH.3033, PNP.4860, KIAA0391.9692, PPA2.27068, COQ2.27235, OXSM.54995, MUDENG.55745 | NA |

The survival outcome is simulated based on a Cox proportional hazards model with three causal genes. The intercept $\beta_{0}= -3.2$ ensures the event rate is 0.25 to match with the colorectal cancer data. Sample size was set as n=200. The total number of the observed genes was set as G=1000.

**Table S2: Simulation Scenarios of Simulation Study C with Smaller Effect Sizes**

| Scenarios | Label | Sample size | # of genes | Event Rate | Sparsity  (# of causal genes/  # of genes) | $\beta_{0}$ | $\beta_{1}$ | $\beta_{2}$ | $\beta_{3}$ |
| --- | --- | --- | --- | --- | --- | --- | --- | --- | --- |
| S1 | n200_G1200_er0.3 | 200 | 1200 | 0.3 | 30/1200 | -2.4 | -2 | -1 | 3 |
| S2 | n200_G1200_er0.5 | 200 | 1200 | 0.5 | 30/1200 | -1.6 | -2 | -1 | 3 |
| S3 | n200_G1200_er0.7 | 200 | 1200 | 0.7 | 30/1200 | -0.7 | -2 | -1 | 3 |
| S4 | n200_G600_er0.3 | 200 | 600 | 0.3 | 30/600 | -2.4 | -2 | -1 | 3 |
| S5 | n200_G600_er0.5 | 200 | 600 | 0.5 | 30/600 | -1.6 | -2 | -1 | 3 |
| S6 | n200_G600_er0.7 | 200 | 600 | 0.7 | 30/600 | -0.7 | -2 | -1 | 3 |
