# Supplementary material for: A robust ensemble feature selection approach to prioritize genes associated with survival outcome in high-dimensional gene expression data
Source: Front Syst Biol. 2024 Mar 21;4:1355595. doi: 10.3389/fsysb.2024.1355595 (PMC11786965; doi:10.3389/fsysb.2024.1355595)

Supplementary Figure 1

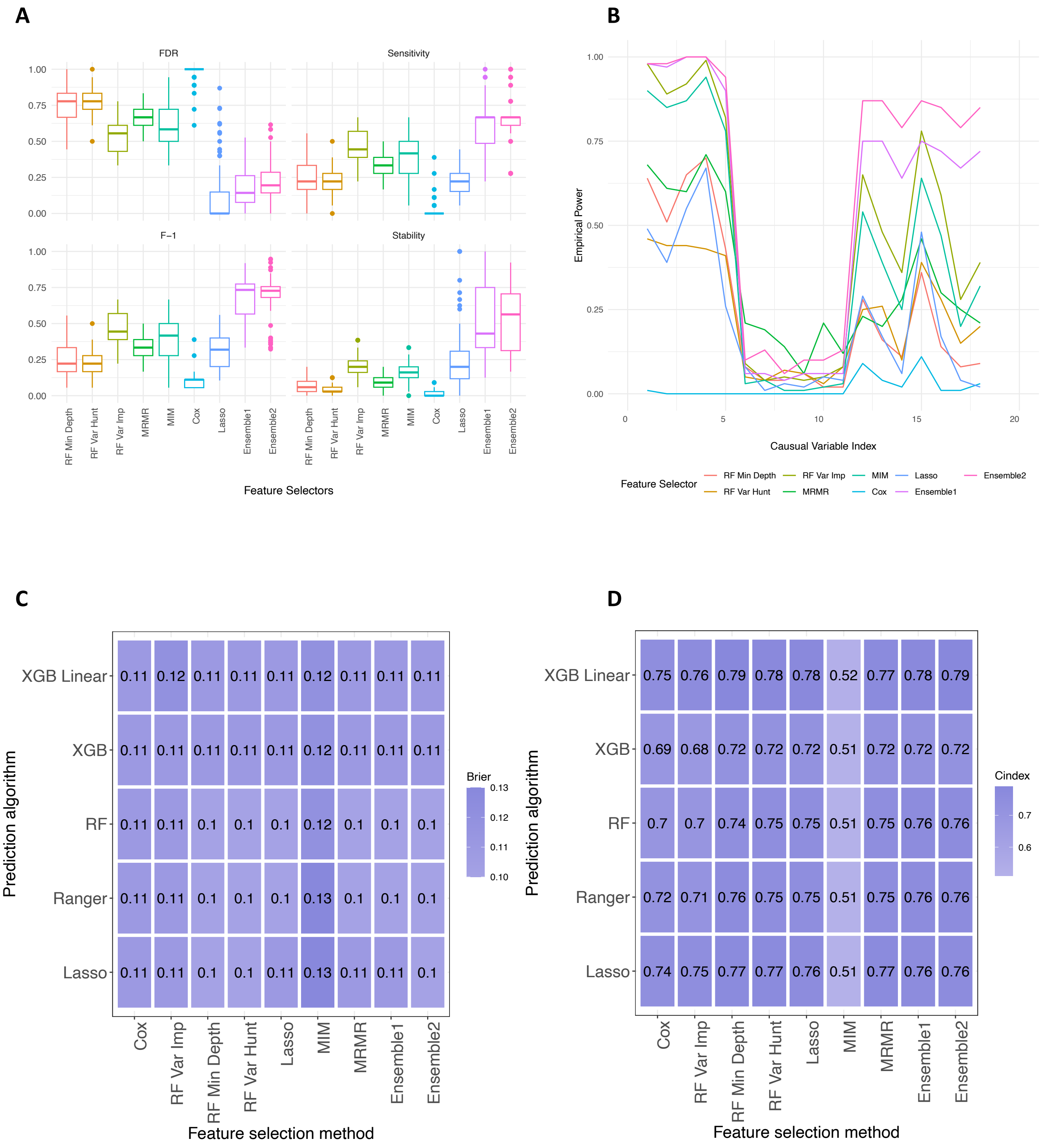

Supplementary Figure 2

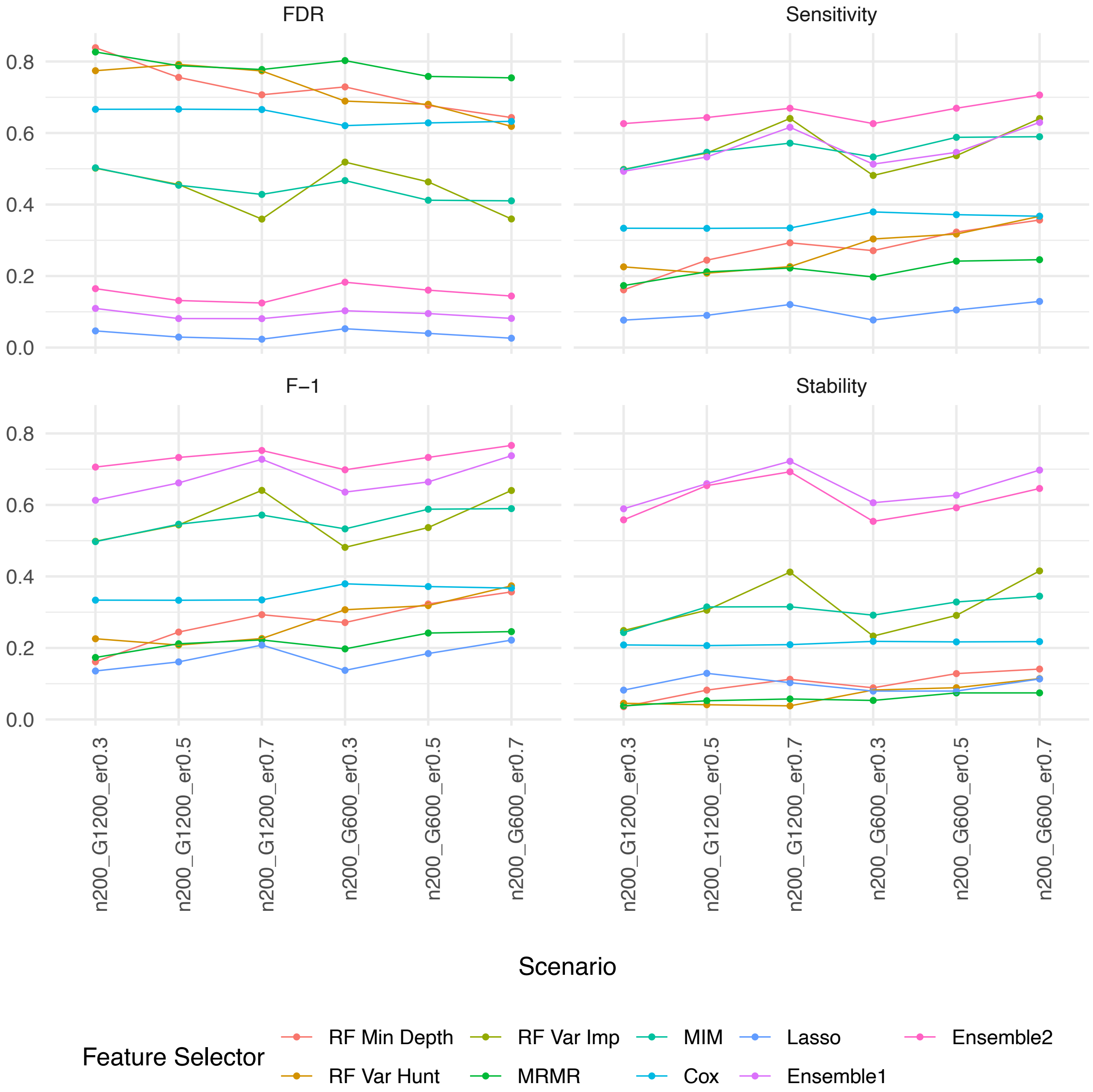

Supplementary Figure 3

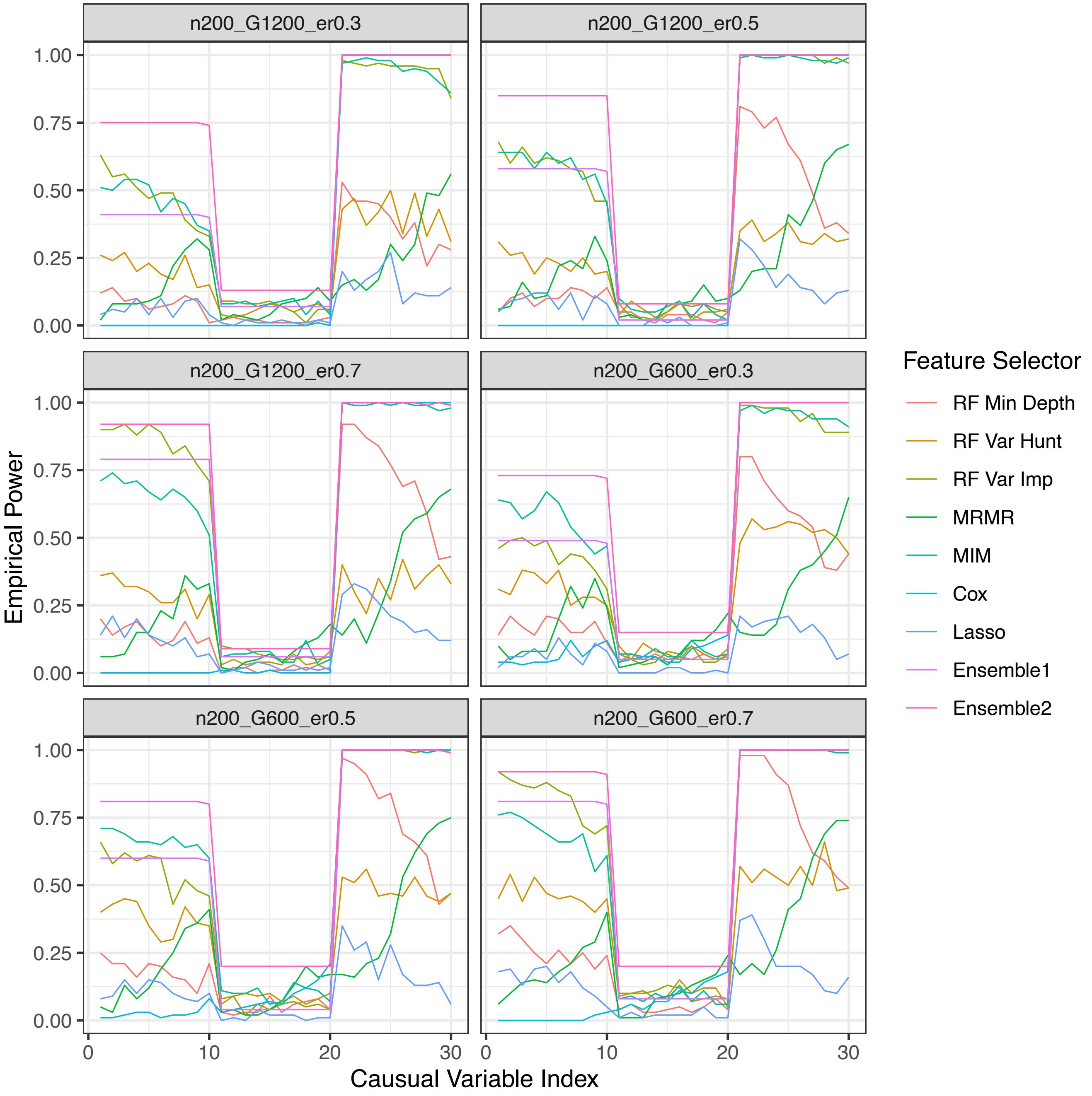

Supplementary Figure 3

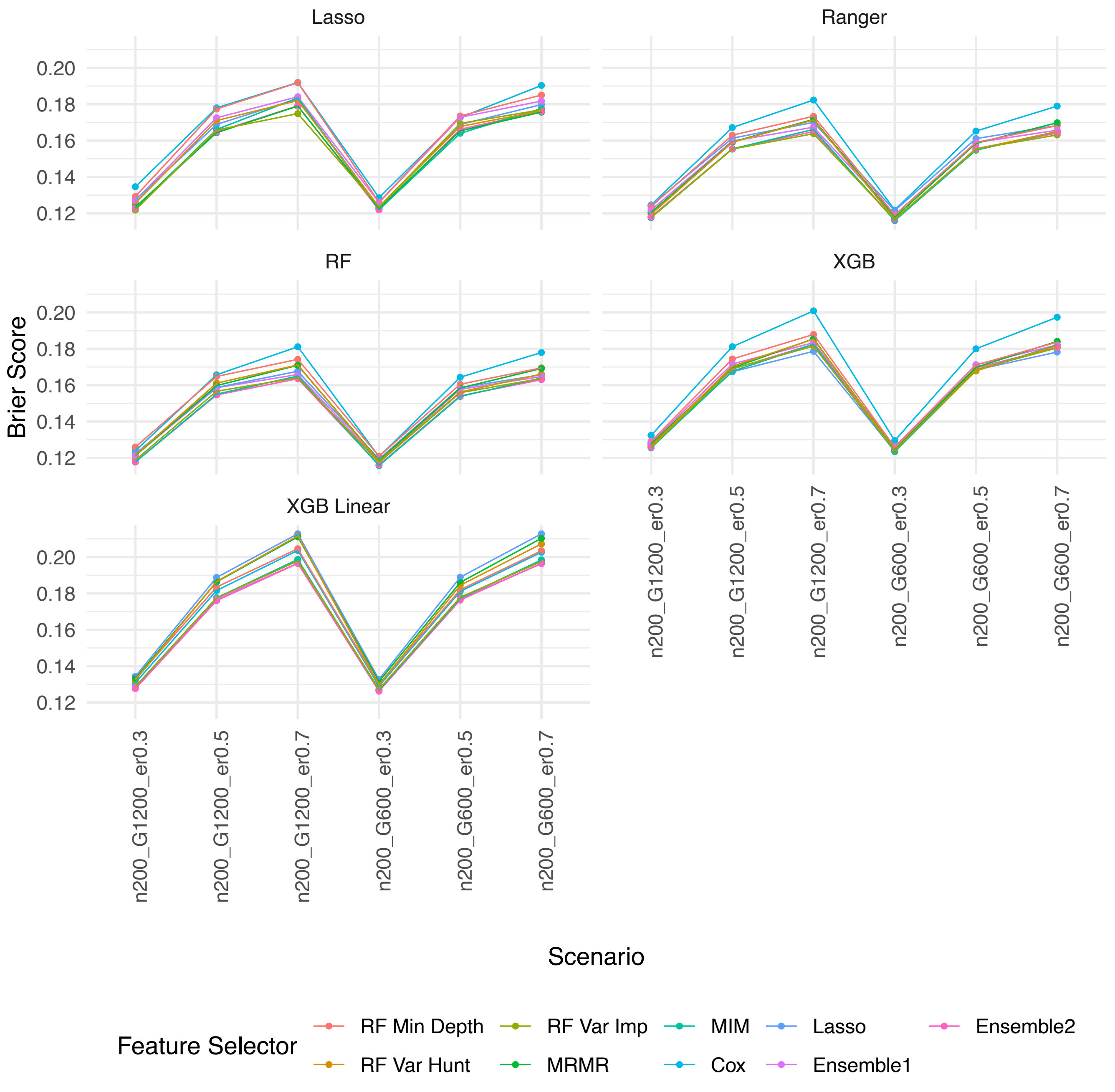

Supplementary Figure 4

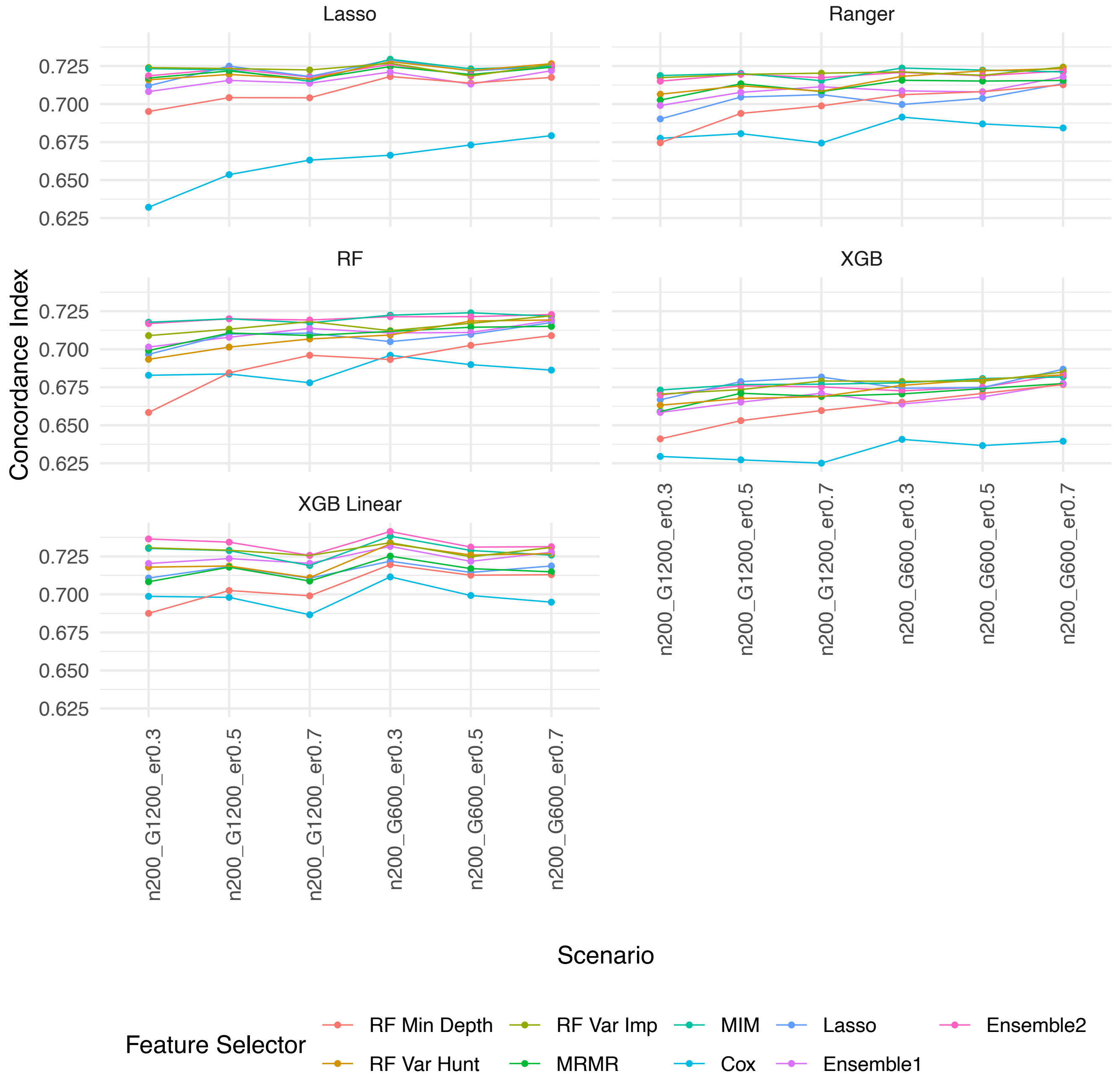

Supplement: Supplementary file 2 [file Image1.PDF]
